# Should practice and policy be revised to allow for risk-proportional payment to human challenge study participants?

**DOI:** 10.1136/medethics-2020-106900

**Published:** 2020-11-05

**Authors:** Euzebiusz Jamrozik, Michael J Selgelid

**Affiliations:** 1 Monash Bioethics Centre, Monash University, Clayton, Victoria, Australia; 2 Ethox & Wellcome Centre for Ethics and Humanities, University of Oxford, Oxford, United Kingdom; 3 Royal Melbourne Hospital Department of Medicine, University of Melbourne, Melbourne, Victoria, Australia

**Keywords:** research ethics, clinical trials, ethics, public health ethics

Human infection challenge studies (HCSs) provide illuminating case studies for several ongoing debates in research ethics, including those related to research risks and payment of participants. Grimwade *et al* 1 add to previous public engagement, qualitative evidence and philosophical literature on these topics.^1–8^ The authors advocate revision of research payment policy and practice based on their main finding that members of the public endorse ex ante payment of participants proportional to research-related risk exposure, in addition to post hoc compensation for any lasting harms that occur.^1^

Although ‘payment for risk’ would diverge from most current research ethics guidelines, it is noteworthy that the difference in payment to participants that their framework would allow might only be small, at least in the case of currently accepted studies. The absolute difference would likely be small because the risk of currently accepted HCS participation (after risk minimisation strategies) is usually very low.^9 10^ On a ‘value of statistical life’ approach, mirroring actuarial accounting for fatal risks, a 1 in 100 000 risk of death might attract an extra payment of only US$96.^1^ Most HCS do not involve fatality risks this high, although, on pessimistic estimates, the risk of COVID-19 HCS in young healthy adults might involve similar levels of risk.^11^ Such risks might arguably be justified by large expected public health benefits,^12^ ^9 11 13^ but it remains controversial whether higher levels of payment could make participant exposure to (greater) risk more acceptable.^10^

HCS participation (in high-income countries) frequently attracts payment of thousands of dollars (ie, many times proposed payments for risk of around US$100). Such payments are usually considered to be justified by the burdens of participation (although the authors note that some HCS perhaps provide inadequate compensation for burdens, eg, total remuneration of £1/hour).^10^ These burdens include large time commitments, prolonged isolation, psychological stress and multiple—sometimes invasive—medical procedures. This raises philosophical questions about what the difference might be, if any, between (payment for) burdens and risks. On one plausible view, what matters is harm to participants—and the probability, severity, duration and reversibility of such harms. Burdens or risks are thus harms that differ in degree along one or more of these features.^14^ We therefore agree with Grimwade *et al* 1 that there is something deeply problematic, if not incoherent, with status quo payment practices and policies.

Additional payment for risk might be concerned primarily with fatality risks or potential lasting harms. Although there have been no deaths in modern HCS and lasting harms are rare,^10^ many challenge designs are associated with small risks of death or lasting harm. There may also be significant uncertainty (eg, the potential for unexpected harm) especially in HCS involving novel or neglected pathogens. Regarding fatality risks, challenge studies that do not fully isolate participants involve small risks that participants will abscond and that even a treatable infection like malaria could become fatal.^10^ Infection with many otherwise mild pathogens is associated with a small probability of lasting harms, such as Guillain-Barré and other postinfectious syndromes, which are not entirely preventable or remediable with treatment.^10^ As a comparator, phase I drug trials involve significant uncertainty and have been rarely associated with death,^15^ severe permanent impairments and individual post hoc compensation for harm of up to £2 million.^16^ As Grimwade *et al* 1 note, payment for risk should not obviate the need to provide compensation for harm, and some might worry that small risk payments would indeed be an inadequate replacement in cases where significant harms end up occurring.

If payment for risk is an inadequate solution for harm and results in minimal change to current payment levels, one might question the justification for a change in practice, especially since higher payments (and perhaps especially payments for risk) are associated with several potential concerns (discussed further below). Grimwade *et al* 1 argue that some such concerns—including those based on one conception of undue inducement, that is, that payment distorts participants’ judgement about risks—are misconceived.

We reported the findings of a project^10^ involving interviews with experts from multiple countries, which identified a wider range of concerns about participant payment. There are conceptions of undue inducement according to which potential problems with high payment include the danger that it could lead participants to conceal important aspects of their medical history (which, contra Grimwade *et al*,^1^ may not always be detectable with basic physiological testing) and/or their simultaneous/recent participation in another paid study (10 p 74). Such omissions might not only increase risks to participants but also undermine the scientific validity of the findings derived from their participation. The issue of ‘overvolunteering’ might in some cases be made worse, rather than improved, by higher levels of payment insofar as money incentivises people to (over-)volunteer. We found that this was particularly a concern in situations of social deprivation (whether in low-income countries or poor communities in high-income countries). The problem of ‘overvolunteering’ might be at least be partially addressed through establishing and enforcing specific regulations.^17^

Moreover, people from different cultural backgrounds may hold different attitudes towards payment. Our study found that significant payment (even for burdens) is not widely accepted in Latin America and has led to controversy in Kenya, for example.^10^ Even if higher levels of payment might be fair to participants, such practices might lead to greater suspicion in the wider community or be proscribed by cultural norms. It would thus be fruitful to explore the acceptability of a framework of payment for risk in different communities. In our view, community acceptance is a necessary condition of the ethical acceptability of research involving human subjects.^10^

Finally, normalising large payments to participants might result in subtle changes in the relationships between researchers and participants. Whereas HCS investigators, who are frequently clinicians, report a strong sense of responsibility for the welfare of the participants in their studies,^10^ commercialisation of early-phase pharmaceutical testing has been associated with deeply problematic practices.^18^ Given the importance of maintaining public trust in HCS and research in general, we should be wary of changing payment practices (perhaps especially in more controversial study designs) in a way that would reduce the tendency of research staff to treat participants with the utmost respect—not only as individuals making a valuable contribution to the generation of scientific knowledge but also as agents who are ends in themselves, rather than merely a means to this knowledge.
